# Al- and Ga-doped graphitic carbon nitride as a temozolomide nanocarrier platform: a DFT study of adsorption and interfacial interactions

**DOI:** 10.1039/d6na00001k

**Published:** 2026-06-05

**Authors:** Maisha Yousuf, Mohammed Sakib Musa, Arafat Mahamud Bhuiyan, Monir Uzzaman, Md. Moazzam Hossain, Kamol Dey

**Affiliations:** a Department of Applied Chemistry and Chemical Engineering, University of Chittagong Chittagong 4331 Bangladesh mohammedsakibmusa@gmail.com kamoldey@cu.ac.bd; b Department of Chemistry, Bangladesh University of Engineering and Technology Dhaka 1000 Bangladesh; c Department of Applied Chemistry, Graduate School of Engineering, Mie University Tsu Mie 514-8507 Japan monircu92@gmail.com; d Bio-Nanomaterials and Tissue Engineering Laboratory (BNTELab), Department of Applied Chemistry and Chemical Engineering, Faculty of Science, University of Chittagong Chittagong 4331 Bangladesh

## Abstract

The rapid degradation and limited tumor accumulation of temozolomide (TMZ) remain important challenges in glioblastoma chemotherapy, motivating the development of nanocarrier systems that can improve TMZ retention and delivery. This study employed density functional theory (DFT) to evaluate the adsorption potential of two-dimensional graphitic carbon nitride (gCN) and its Al/Ga-doped variants (gCN-Al and gCN-Ga) as nanocarriers for TMZ delivery. A comprehensive analysis, including the electronic structure, natural bond orbital, quantum theory of atoms-in-molecules, and noncovalent interaction analyses, revealed that TMZ adsorbs onto the nanocarriers *via* spontaneous, physisorptive interactions, primarily by hydrogen bonding and van der Waals forces. The adsorption strength follows the order gCN-Ga > gCN-Al > pristine gCN, with gCN-Ga exhibiting the most favorable adsorption energy (−1.24 eV). Doping introduces new electronic states that narrow the HOMO–LUMO gap and enhance charge transfer, rationalizing the improved adsorption. The absence of imaginary frequency confirmed that each optimized geometry corresponds to a true minimum on the potential energy surface. Thermodynamic property analyses revealed the spontaneous and exothermic nature of the drug–nanocarrier complex formation. Recovery-time estimates suggest that TMZ desorption is thermally accessible, with Ga doping producing the longest predicted residence time. These results suggest that Al/Ga doping can modulate TMZ–gCN interactions at the molecular level and may provide a useful computational basis for future experimental evaluation of gCN-based TMZ delivery platforms.

## Abstract 2

Al/Ga doping strengthens temozolomide adsorption on gCN *via* charge transfer, hydrogen bonding, and van der Waals interactions: insights from FMO, NBO, NCI, and QTAIM analyses.

## Graphical abstract

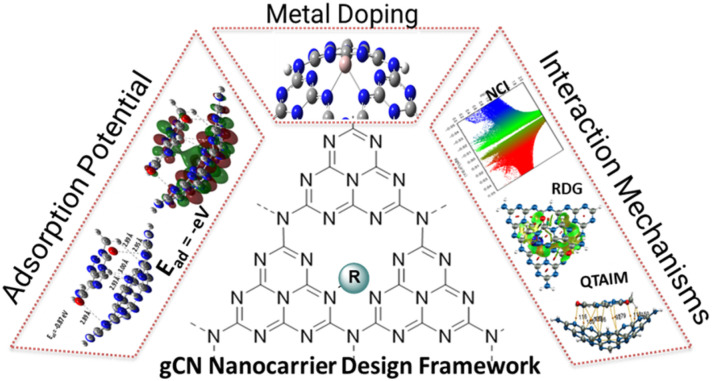

## 1 Introduction

Glioblastoma (GB) represents approximately 45.6% of all primary malignant brain tumors and is distinguished by its marked aggressiveness, high rate of recurrence, and pronounced heterogeneity.^1^ The standard clinical management involves maximal surgical resection, followed by adjuvant radiotherapy in combination with chemotherapy using temozolomide (TMZ).^2^ TMZ is commonly employed as the first-line chemotherapeutic agent for glioblastoma. It is a lipophilic imidazotetrazine derivative of the alkylating agent dacarbazine, originally developed in the 1970s for melanoma therapy. Since 2005, it has become the standard chemotherapeutic agent for glioblastoma (GB), primarily due to its exceptional oral bioavailability (∼98%) and its remarkable ability to cross the blood–brain barrier (BBB).^2^ However, its clinical efficacy is hindered by the development of chemoresistance and the occurrence of multiple adverse effects.^1^ Given the significant adverse effects associated with TMZ and the challenge of chemoresistance, current multimodal therapeutic strategies remain insufficient in enhancing clinical outcomes for glioblastoma patients.

Despite its clinical utility, oral TMZ therapy faces several pharmacokinetic and therapeutic limitations that may be addressed through nanodelivery strategies.^3^ Although TMZ can cross the BBB, only a limited fraction of systemically administered TMZ is expected to reach the tumor site in an active form, which can restrict therapeutic efficacy.^4^ Nanocarriers can be strategically designed to traverse the BBB *via* multiple transport mechanisms such as cell-mediated transcytosis.^5,6^ Again, to attain therapeutic levels of TMZ within the brain, administration of high systemic doses is frequently necessary,^4^ which is associated with considerable systemic toxicity, including hematological adverse effects such as neutropenia,^7^ thrombocytopenia,^7,8^ and lymphopenia, as well as fatigue, nausea, and vomiting.^9^ Various nanodelivery systems have been developed to reduce the required TMZ dose and minimize exposure to healthy tissues by selectively targeting tumor cells and enhancing drug accumulation at the tumor site thereby lowering systemic side effects.^4,10–12^ Furthermore, TMZ has a very short half-life (about 2 hours) in the bloodstream, rapidly breaking down into inactive compounds before it can reach the tumor.^13^ Encapsulation within a nanoparticle protects TMZ from this rapid degradation in the systemic circulation.^13^ Also, nanocarriers can be engineered to release TMZ in a controlled and sustained manner at the tumor site, maintaining therapeutic concentrations over a longer period and improving treatment efficacy.^10^

Graphitic carbon nitride (gCN), a two dimensional π-conjugated organic polymer, has been extensively investigated as a metal-free photocatalyst for hydrogen evolution,^14,15^ degradation of pollutants, reduction of CO_2_, synthesis of H_2_O_2_, and other solar-driven reactions,^16^ primarily due to its visible-light activity, nitrogen-rich framework, and tunable electronic structure. Several strategies have been implemented to enhance charge separation, surface reactivity, and photocatalytic efficiency of gCN-based systems by elemental doping, defect engineering, or heterojunction construction.^17^ Beyond photocatalysis, gCN has attracted interest as a two-dimensional platform for molecular adsorption and drug–carrier design because of its chemical stability, nitrogen-rich surface, and tunable electronic structure. Some DFT studies suggest its potential in drug delivery systems for melphalan,^18^ cisplatin,^19^ carboplatin,^20^ curcumin,^21^ hesperetin,^22^ flutamide,^23^ lonidamine,^24^ levosimendan,^25^ and doxorubicin^26^ adsorption. However, experimental validation of gCN-based TMZ delivery remains limited, and, to the best of our knowledge, Al/Ga-doped gCN has not been examined for TMZ adsorption by either DFT or experimental approaches.

Introducing dopants into nanocarriers can modify their drug adsorption properties.^27^ The existing literature demonstrates the influence of the doping process on drug delivery system.^28,29^ This deliberate modification to the host nanocarriers leads to a substantial increase in the nanocarrier's adsorption potential.^30,31^ In this work Al and Ga atoms were introduced in a gCN framework to tailor the surface electronic structure to enhance TMZ adsorption. A comparative DFT study of pristine gCN, Al doped gCN and Ga doped gCN as molecular models of TMZ nanocarrier interfaces is presented. The key novelty is the ability to correlate the dopant induced electronic modulation with the adsorption strength, the type of the interaction and the estimated desorption behavior. The adsorption energetics of the drug–carrier interface were coupled with frontier molecular orbital (FMO) and density of states (DOS) analyses, conceptual DFT, natural bond orbital (NBO) analysis, noncovalent interaction (NCI) and quantum theory of atoms in molecules (QTAIM) analysis to gain atomistic insight into the drug–carrier interface. Furthermore, TD-DFT computations were undertaken to investigate the UV-visible absorption response of the isolated systems, as well as complexed ones. Frequency calculations were performed to ensure that the optimized geometries are indeed true minima of the potential energy surface. Thermodynamic parameters were evaluated and estimation of the TMZ recovery time was performed to assess the favorability and reversibility of TMZ adsorption. This integrated approach allows a molecular-level understanding of the interaction between TMZ and gCN and serves as a guide for the rational design of nanocarrier platforms based on doped gCN.

It was found that TMZ is adsorbed spontaneously on all types of gCN, with the main forces being hydrogen bonding and van der Waals. It was discovered that doping with Ga and Al, which had the strongest effect, increased the adsorption energy. Electronic structure analyses confirm that doping introduces new states that reduce the HOMO–LUMO gap, facilitating stronger interactions. These computational insights suggest that Al/Ga-doped gCN can serve as a useful platform for TMZ adsorption and provide a basis for future experimental evaluation of gCN-based TMZ drug delivery systems in glioblastoma therapy.

## 2 Computational methods

All quantum chemical calculations were conducted with Gaussian 09,^32^ employing the B3LYP^33,34^ hybrid exchange–correlation functional in combination with the 6-31+G(d,p) Pople split-valence basis set.^35–37^ The B3LYP functional integrates Becke's 1988 (ref. 38) exchange functional with the Lee–Yang–Parr correlation functional and incorporates the local density approximation (LDA) for correlation effects.^39^ Owing to its balance between accuracy and computational efficiency, B3LYP is widely used across computational chemistry. However, it exhibits known deficiencies in describing long-range dispersion forces.^40^ To address this limitation, Grimme's dispersion correction (DFT-D3) with the Becke–Johnson damping (D3(BJ)) approach was employed, which improved the treatment of van der Waals interactions, particularly at medium and short interatomic distances.^40,41^ This correction enhances the accuracy of interaction energies in noncovalent systems. Water, being the primary biological solvent, was modeled using the polarizable continuum model (PCM), thereby accounting for the solvent effect of water.^42^ Vibrational frequency calculations were carried out at the same level of theory to verify that each optimized structure represents a true minimum on the potential energy surface. Time-dependent DFT (TD-DFT) calculations were performed at the same theoretical level to evaluate electronic absorption spectra.

The adsorption energy (*E*_ads_) of the TMZ on NCs (gCN, gCN-Al, gCN-Ga) was estimated using

1 *E*_ads_ = *E*_TMZ@NC_ − *E*_NC_ − *E*_TMZ_

where *E*_TMZ@NC_, *E*_NC_ and *E*_TMZ_ are the energies of the TMZ@gCN, TMZ@gCN-Al, and TMZ@gCN-Ga complexes and the individual gCN, gCN-Al, gCN-Ga and TMZ respectively.

The energies of the highest occupied molecular orbital (*ε*_H_) and the lowest unoccupied molecular orbital (*ε*_L_) were utilized to calculate conceptual DFT based reactivity indices, including the HOMO–LUMO energy gap (*E*_gap_), chemical potential (*µ*), hardness (*η*), softness (*S*), and electrophilicity (*ω*) using the formulae proposed by Janak *et al*.^43^ and Parr *et al.*^44^ These indices provide insights into the electronic structure and chemical reactivity of the studied systems. These conceptual DFT reactivity descriptors are extensively employed in the scientific literature^45,46^ and are calculated using the following relationships:

2 *E*_gap_ = *ε*_L_ − *ε*_H_

3
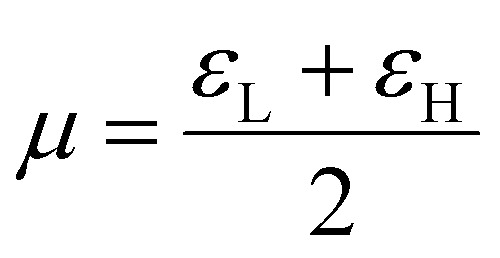
4
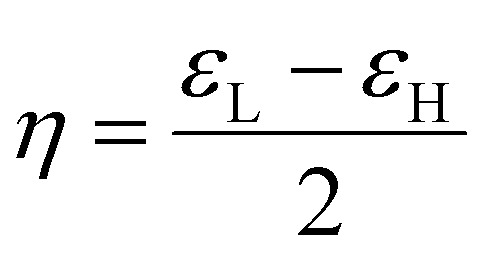
5
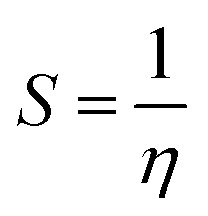
6
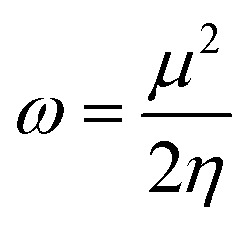


In addition, interactions within the complexes were analyzed using thermodynamic parameters such as Gibbs free energy (Δ*G*), enthalpy (Δ*H*), and entropy (Δ*S*). These values were calculated using the following equations:

7 Δ*M* = *M*_TMZ@NC_ − *M*_NC_ − *M*_TMZ_

8
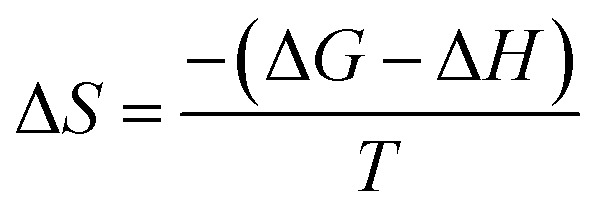


As explained in the above formula, Δ*M* refers to the Δ*G* and Δ*H* energies for the optimized complexes. Δ*S* was calculated at 298.15 K. Besides, *M*_TMZ@NC_, *M*_TMZ_, and *M*_NC_ define the *G*/*H* parameters of the optimized complexes, TMZ, and nanocarriers, respectively. The recovery time (*τ*) was calculated to determine how likely TMZ is to detach from the nanocarriers following the transition theory:9
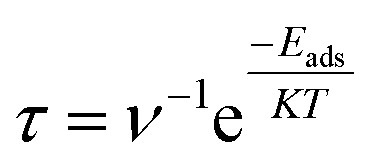
where *ν* is the frequency constant (10^18^ s^−1^), *k* is Boltzmann's constant (8.62 × 10^−5^ eV K^−1^) and *T* is temperature with values of 298.15 K (room temperature), 310.15 K (normal body temperature) and 315.15 K (tumor microenvironment).^47,48^

Additionally, Natural Bond Orbital (NBO)^49^ analysis was carried out to offer a simplified perspective on drug–nanocarrier interactions by examining the electron donation from Lewis's base-type orbitals (electron donors) to Lewis's acid-type orbitals (electron acceptors) which is useful to interpret results. This analysis helped gain a more comprehensive understanding of the computational findings and was carried out using the NBO 3.1 program. The electronic structures of TMZ@NCs were further analyzed by computing the density of states (DOS) and partial density of states (PDOS) using the Multiwfn software.^50^

To characterize the bonding nature within the investigated TMZ@NC complexes, the quantum theory of atoms in molecules (QTAIM), formulated on Bader's^51,52^ topological analysis of the electron density (*ρ*(*r*)) was employed. The QTAIM approach enabled the identification of bond paths (BPs) and bond critical points (BCPs) between interacting fragments (TMZ and NCs). At each BCP, critical topological descriptors were extracted, including electron density (*ρ*(*r*)), kinetic energy density (*G*(*r*)), local potential energy density (*V*(*r*)), electron Hamiltonian energy density *H*(*r*), the Laplacian of the electron density (∇^2^*ρ*(*r*)), and the bond ellipticity (*ε*). These parameters distinguish covalent or ionic bonds (*ρ*(*r*) > 0.1 a.u.) from noncovalent interactions such as van der Waals forces (*ρ*(*r*) < 0.1 a.u.). Complementary to this, the NCI index was applied to map and visualize noncovalent interactions. 2D and 3D NCI isosurfaces were rendered and color-coded according to (*λ*_2_)*ρ* values within the range of −0.05 a.u. (blue, strong attractive interactions) to 0.05 a.u. (red, repulsive interactions).^53^ All QTAIM and NCI computations were carried out using Multiwfn 3.8,^50^ and graphical representations were generated with Visual Molecular Dynamics (VMD).^54^ Because the calculations were performed on a hydrogen-passivated finite gCN fragment, the results primarily describe local TMZ–surface interactions rather than adsorption on extended or defect-rich polymeric gCN. Periodic calculations or larger cluster models would be required to evaluate long-range electronic effects, surface heterogeneity, and coverage-dependent adsorption.

## 3 Results and discussion

### 3.1 Optimized geometries and adsorption energetics

**Fig. 1:**
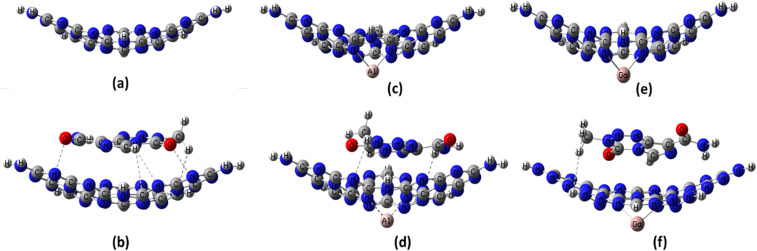
Optimized structures of (a) gCN, (b) TMZ@gCN, (c) gCN-Al, (d) TMZ@gCN-Al, (e) gCN-Ga and (f) TMZ@gCN-Ga.

The computational model of graphitic carbon nitride (gCN) in this study was built from three heptazine units, containing a total of 18 carbon, 26 nitrogen, and 8 hydrogen atoms. To mitigate boundary effects and represent a non-periodic fragment, all peripheral atoms of the model were passivated with hydrogen.^55^ Many previous studies have demonstrated that such an aperiodic structure could also accurately reflect the properties of gCN.^55–57^ Consistent with prior reports identifying the cavity between the three heptazine units as the energetically preferred site, metals were doped in the hollow position,^58–60^ forming a moiety composed of 2 nitrogen atoms and a metal atom. The optimized structures (Fig. 1 and the S1) show the metal atom displaced from the planar base of the gCN, inducing measurable deformation of the gCN monolayer – a result aligning with previous findings.^55,61^ The experimental conformation of a carrier utilized for drug delivery is governed by factors such as surface area, morphology, pH, ionic strength, temperature, and non-covalent interactions. These complexities are difficult to capture fully with quantum-chemical methods. Therefore, to provide foundational insight into drug–carrier binding, this study employed a simplified gCN model comprising three heptazine units. This fragment captures local steric and electronic effects more realistically than a single heptazine unit, while remaining computationally feasible. Although extended polymeric networks in real gCN impose additional conformational constraints on drug binding, the present study is necessarily limited to this tri-heptazine model.

The calculated adsorption energies and interfacial nearest atom pair distances for TMZ on nanocarriers are summarized in Table 1. Adsorption strength increases with metal doping, from −0.88 eV (gCN) and −0.97 eV (gCN-Al) to −1.24 eV (gCN-Ga). This trend aligns with a correlated decrease in the closest atom pair distance of 2.60 Å, 2.51 Å and 2.15 Å respectively.

**Table 1:** Calculated adsorption energy in eV with the nearest adsorbing atom distance of the TMZ@nanocarrier complexes

Complexes	*E*_ads_ (eV)	Distance
TMZ@gCN	−0.88	H69⋯N39 (2.60 Å)
TMZ@gCN-Al	−0.97	H72⋯N11 (2.51 Å)
TMZ@gCN-Ga	−1.24	H72⋯N39 (2.15 Å)

The nearest interacting atoms involve hydrogen from TMZ and nitrogen from the nanocarriers. In the most stable system (TMZ@gCN-Ga), the contact occurs between H72 (from the amine group of TMZ) and N39 (nitrogen from gCN-Ga). The shorter distance and stronger adsorption in the doped variants suggest a more effective intermolecular interaction relative to the pristine counterpart (gCN).

The closest contact and probable propensity for hydrogen bonding between the TMZ/nanocarrier atoms is influenced by electronegativity. The H72⋯N39 adsorption distance for the Ga-doped system benefits from the higher polarity of the amine hydrogen, enhancing its partial positive charge and strengthening the electrostatic attraction to the electronegative nitrogen (N39) from the heptazine framework. A similar trend is observed for the gCN-Al adsorption distance (H72⋯N11). Conversely, this effect appears less pronounced in TMZ@gCN when the interacting hydrogen (H69) originates from a less polar methyl group, increasing the adsorption distance (2.60 Å).

### 3.2 Molecular electrostatic potential surface analysis

**Fig. 2:**
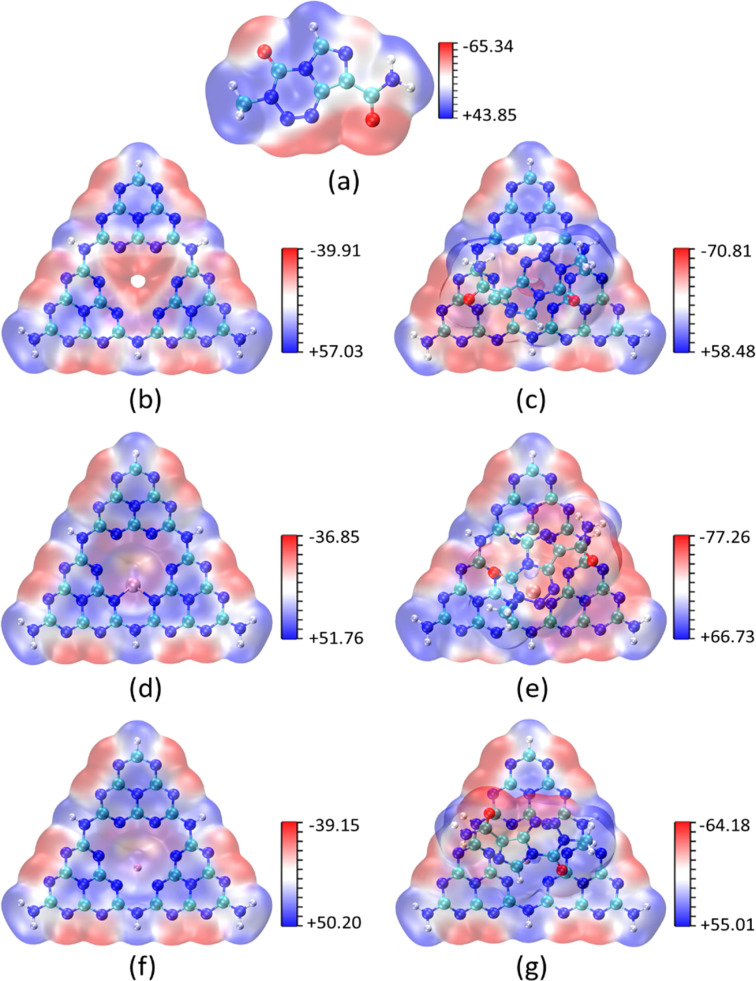
Electrostatic potential energy surface (ESP) of optimized (a) TMZ, (b) gCN, (c) TMZ@gCN, (d) gCN-Al, (e) TMZ@gCN-Al, (f) gCN-Ga and (g) TMZ@gCN-Ga. The red and blue points represent the local minima and maxima points of the ESP surface, respectively, calculated in kcal mol^−1^.

The electrostatic potential (ESP) surfaces (Fig. 2) of TMZ, nanocarriers, and their corresponding complexes were analyzed to elucidate the charge-complementary interactions governing TMZ adsorption. The TMZ molecule exhibits a pronounced charge separation, with its global minimum (−65.34 kcal mol^−1^) located on the carbonyl oxygen atoms and the global maximum (+43.85 kcal mol^−1^) localized on hydrogen-bearing sites. This distinct polarity defines TMZ's nucleophilic and electrophilic regions. The surface of the pristine gCN, on the other hand, has a range distribution of ESP values from −39.91 to +57.03 kcal mol^−1^ across its heptazine units, with the global minima positioned at the hollow site, introducing a number of potential binding sites. The ESP profile of the nanocarriers was significantly modified by the integration of Al and Ga dopants. Doping creates localized electron deficient regions around the metal sites that become electrophilic. This is consistent with the well-known role of atomic-scale defect engineering, where such local polarization results in ideal adsorption sites for molecules.^62^ The resulting charge asymmetry enhances the surface's electrostatic complementarity with the TMZ molecule. Upon TMZ adsorption, all complexes exhibited a substantial broadening of their ESP ranges compared to their isolated components. The most significant expansion was observed for the TMZ@gCN-Al complex (−77.26 to +66.73 kcal mol^−1^), indicating the strongest interfacial charge redistribution and polarization response. The widening of the ESP range upon complexation indicates interfacial polarization and supports the role of electrostatic complementarity in TMZ adsorption, where electron-rich (carbonyl oxygens) and electron deficient (amine hydrogen) regions of TMZ align with their complementary areas on the nanocarrier.^63^

### 3.3 Frontier orbital distribution and density of states

**Fig. 3:**
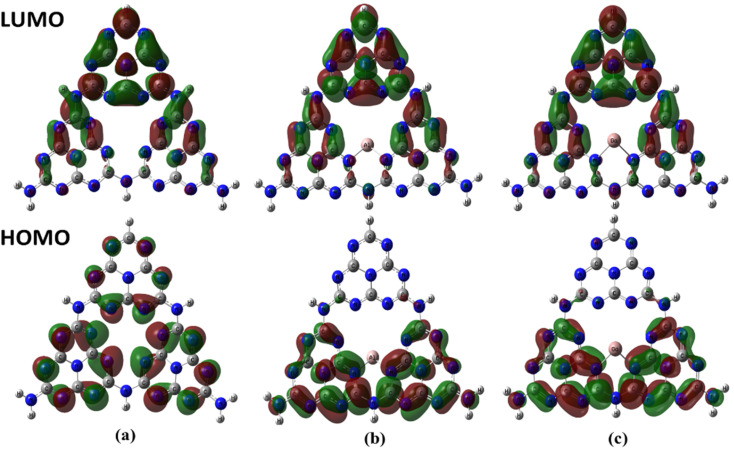
Top view of HOMO–LUMO orbitals of (a) gCN, (b) gCN-Al, and (c) gCN-Ga.

The energy gap between the highest occupied molecular orbital (HOMO) and the lowest unoccupied molecular orbital (LUMO), along with the density of states (DOS), serves as a valuable tool for investigating charge transfer and intramolecular energy exchange processes within a molecular system of interest. In the pristine gCN, the HOMO is primarily localized on the nitrogen lone pairs of the heptazine units, while the LUMO resides on the carbon–nitrogen network (Fig. 3). This localized orbital distribution suggests limited intrafragment electronic delocalization within the finite cluster model. Aluminum or gallium doping substantially restructures the HOMO, promoting partial delocalization of nitrogen lone pairs across the C–N bonds and intensifying electron density around the respective dopant coordination sites. The redistribution suggests an enhanced hybridization of the dopant orbital with the nearby gCN framework which increases the HOMO energy from −6.82 eV (gCN) to −3.94 eV (gCN-Al), with the reduction of the HOMO–LUMO gap. The energy level diagram (Fig. 5) of gCN exhibits a HOMO–LUMO gap of 3.77 eV. Doping dramatically reduces this gap to 0.94 eV (gCN-Al) and 0.89 eV (gCN-Ga) due to the doping-induced elevation of the HOMO level while LUMO energy largely remains unchanged.

A notable difference emerges between the computed electronic gap (*ε*_gap_) in this work and the established band gap of bulk gCN (∼2.7 eV).^64^ The computational model employed here, which utilizes a finite cluster of the three heptazine units without periodic boundary conditions, yields a HOMO–LUMO gap of 3.77 eV. This elevation is a recognized artifact of the finite-cluster approximation, where quantum confinement in a limited molecular fragment artificially enlarges the gap relative to the extended periodic material. Similar enlargement was documented in prior DFT studies using comparable cluster models and the B3LYP functional.^19,21^

**Fig. 4:**
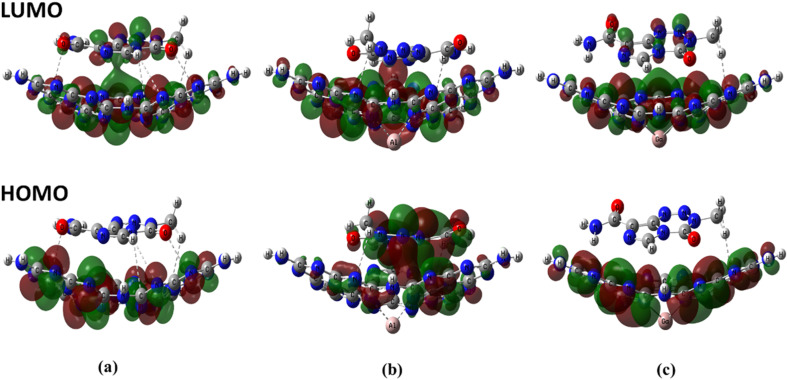
Lateral view of HOMO–LUMO orbitals of (a) TMZ@gCN, (b) TMZ@gCN-Al, and (c) TMZ@gCN-Ga.

**Fig. 5:**
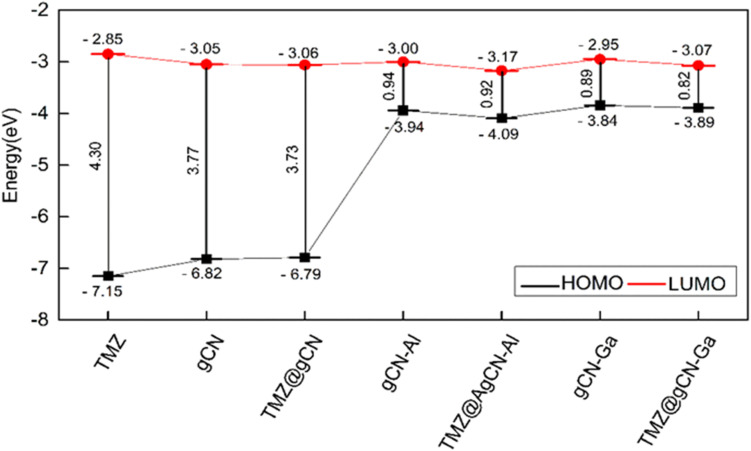
HOMO–LUMO energy level diagram.

Again, in the pristine TMZ@gCN complex, similar to the gCN, the HOMO is fully localized over the nitrogen lone pairs of the gCN's heptazine units (Fig. 4(a) and S2(a)), while the LUMO is delocalized over the conjugated C–N bonds of the heptazine framework with a noticeable contribution from the adsorbed TMZ molecule. This results in charge transfer between TMZ and gCN confirming the interaction between them. Formation of the complex reduces the HOMO–LUMO gap to 3.73 eV. This slight narrowing is attributed to a minor uplift of the HOMO, while the LUMO remains unchanged and identical to that of pristine gCN (Fig. 5), indicating negligible electronic coupling.

In the TMZ@gCN-Al complex, the HOMO is predominantly localized on the TMZ molecule and the adjacent C–N framework, whereas the LUMO is delocalized across the heptazine network (Fig. 4(b) and S2(b)). This frontier orbital arrangement clearly suggests the possibility of a transfer of electrons. Here, transfer of electrons occurs from TMZ to the carrier, enabling efficient physisorption. After the adsorption on TMZ, the energy gap reduces to 0.92 eV. Here, the primary driver is the stabilization and lowering of the system's LUMO energy (Fig. 5). This LUMO stabilization signifies a more pronounced electronic perturbation compared to the undoped case. A similar orbital distribution is observed in TMZ@gCN-Ga; however, the reversed HOMO/LUMO localization suggests that charge transfer occurs from the carrier to the drug molecule.

**Fig. 6:**
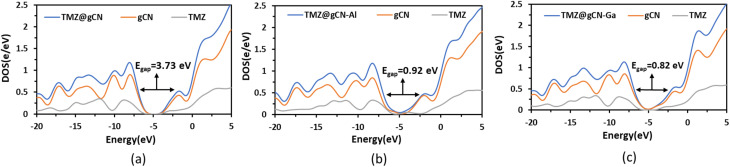
Density of states (DOS) spectra of (a) TMZ@gCN, (b) TMZ@gCN-Al, and (c) TMZ@gCN-Ga.

Critically, across all complexes, the projected DOS (PDOS) of the TMZ and nanocarrier fragments show no orbital overlap and the total DOS intensity remains largely unchanged (Fig. 6). This pattern-where the electronic structure is perturbed without evidence of covalent hybridization-is a hallmark of physisorption, consistent with reports that physisorption often does not substantially alter the host's electronic properties.^65^ The interaction is therefore best described as non-covalent, with doping fundamentally enhancing the carrier's sensitivity to TMZ by enabling LUMO stabilization.

### 3.4 Conceptual DFT-based reactivity descriptors

Frontier molecular orbitals (HOMO and LUMO) determine a molecule's optical and electrical properties. Therefore, the HOMO–LUMO energy gap (*ε*_gap_) is a key indicator for assessing a molecule's kinetic stability, chemical reactivity, and chemical hardness.^66,67^ A molecule with a narrow energy bandgap is a soft molecule due to its high chemical reactivity and low kinetic stability. A reduced energy gap in this method signifies substantial intramolecular charge transfer, which is a key indicator of a molecule's reactivity.^21^

The incorporation of Al/Ga into the gCN framework induces a fundamental electronic reorganization that extends to its drug–carrier complexes. This transformation is quantitatively captured by conceptual density functional theory (DFT) indices (Table 2), which reveal systematic changes in electronic reactivity that are relevant to TMZ adsorption and interfacial polarization.

**Table 2:** Conceptual-DFT indices for the studied systems – HOMO (*ε*_H_ (eV)), LUMO (*ε*_L_ (eV)), HOMO–LUMO gap (*ε*_gap_ (eV)), chemical hardness (*η* (eV)), softness (*S* (eV^−1^), chemical potential (*µ* (eV)), and electrophilicity index (*ω* (eV))

System	*ε*_L_	*ε*_H_	*ε*_gap_	*η*	*S*	*µ*	*ω*
TMZ	−2.85	−7.15	4.30	2.15	0.47	−5.00	5.81
gCN	−3.05	−6.82	3.77	1.89	0.53	−4.94	6.46
TMZ@gCN	−3.05	−6.79	3.73	1.87	0.54	−4.93	6.50
gCN-Al	−3.00	−3.94	0.94	0.47	2.13	−3.47	12.81
TMZ@gCN-Al	−3.17	−4.09	0.92	0.46	2.17	−3.63	14.32
gCN-Ga	−2.95	−3.84	0.89	0.45	2.25	−3.40	12.95
TMZ@gCN-Ga	−3.07	−3.89	0.82	0.41	2.44	−3.48	14.77

Metal doping narrowed the HOMO–LUMO gap of the carrier from ∼3.77 eV to ∼0.9 eV, which directly contributed to the sharp decrease in chemical hardness (*η*) and a four to five-fold increase in global softness (*S*) as well. This softness is preserved and even slightly enhanced in the drug-loaded complexes, TMZ@gCN-Al (*S* = 2.17 eV^−1^) and TMZ@gCN-Ga (*S* = 2.44 eV^−1^). The correspondingly low chemical hardness (*η* ∼ 0.45 eV) implies that these systems can readily undergo electronic reorganization.

Concurrently, doping significantly elevates the electrophilicity index (*ω*). The gCN-Al and gCN-Ga carriers exhibit a *ω* of 12.81 eV and 12.95 eV respectively, which increases upon TMZ adsorption to 14.32 eV and 14.77 eV respectively. The electrophilicity index quantifies the energy stabilization a system gains upon acquiring electron density.^44^ The elevated *ω* signifies a strong thermodynamic driving force for the doped carrier to act as an electron acceptor.^68^

### 3.5 Donor–acceptor interactions from NBO analysis

Natural Bond Orbital (NBO) analysis was performed to quantify the charge transfer (CT) interactions stabilizing the TMZ–nanocarrier complexes (Table 3). The second-order perturbation theory energy (*E*^2^) provides a measure of the donor–acceptor orbital interactions, elucidating the electronic stabilization mechanism upon adsorption.^69^

**Table 3:** The natural bonding orbitals of the studied complexes and their respective stabilization energies (kcal mol^−1^)

Complex	Transition	Donor-NBO	Acceptor-NBO	*E* ^2^
TMZ@gCN	gCN → TMZ	π(C1–N13)	π*(N58–N59)	0.23
		π(C33–N41)	π*(N57–C64)	0.24
		LP(1)N39	σ*(C65–H69)	0.52
		LP(2)N39	σ*(C65–H69)	0.46
		LP(2)N43	σ*(C64–H67)	0.38
	TMZ → gCN	π(O53–C63)	π*(C36–N42)	0.45
		π(O54–C66)	π*(C19–N26)	0.78
		π(N57–C64)	π*(C22–N30)	0.33
		LP(1)O53	π*(C36–N42)	0.46
		LP(1)O54	π*(C19–N26)	0.29
		LP(1)N57	π*(C22–N30)	0.26
TMZ@gCN-Al	gCN-Al → TMZ	π(C1–N10)	LP(1)C65	0.12
		π(C2–N11)	σ*(N61–H72)	0.34
		π(C6–N14)	LP(1)C65	0.14
		π(C20–N24)	π*(N58 – C63)	0.18
		LP(1)N11	σ*(N61 – H72)	0.37
		LP(1)N43	σ*(C66 – H71)	0.20
		LP(2)N43	σ*(C66 – H71)	0.20
	TMZ → gCN-Al	π(O54–C64)	π*(C34–N38)	0.13
		π(N58–C63)	π*(C2–N11)	0.27
		π(N59–C62)	π*(C21–N27)	0.47
		LP(1)N57	π*(C33–N41)	0.38
		LP(1)N58	π*(C3–N8)	0.14
		LP(1)N59	π*(C21–N27)	0.11
		LP(1)N60	π*(C22–N29)	0.16
		LP(2)N60	π*(C22–N29)	1.49
		LP(1)N61	σ*(N16–H47)	0.21
		LP(1)C65	π*(C1–N10)	1.27
	TMZ → Al	LP(1)N56	LP*(2)Al53	0.04
		LP(1)N56	LP*(4)Al53	0.03
		LP(2)N60	LP*(2)Al53	0.03
TMZ@gCN-Ga	gCN-Ga → TMZ	π(C19–N23)	σ*(C66–H69)	0.12
		LP(1)N15	π*(O55–C67)	0.10
		LP(2)N41	LP(1)C65	0.14
	TMZ → gCN-Ga	π(N58–C63)	π*(C35–N39)	0.14
	TMZ → Ga	LP(1)C65	LP(2)Ga53	0.12
		LP(1)C65	LP*(4)Ga53	0.06

For the TMZ@gCN complex, the analysis reveals bidirectional, albeit asymmetric, charge transfer. In the gCN → TMZ direction, weak stabilization (*E*^2^ = 0.23 to 0.52 kcal mol^−1^) occurs *via* donation from the heptazine ring's π-system and nitrogen lone pairs (*e.g.*, LP(N39) → σ*(C65–H69)) into TMZ's antibonding orbitals. A stronger CT is observed in the reverse direction (TMZ → gCN), with *E*^2^ values up to 0.78 kcal mol^−1^ for donations from TMZ's π(O54–C66) and lone pairs (*e.g.*, LP(O53)) into the π*-system of gCN. This indicates a net electron flow from the drug to the carrier, consistent with physisorption dominated by electrostatic and hydrogen-bonding interactions. Doping significantly alters the charge transfer profile and drug adsorption in drug delivery system.^70^ In the TMZ@gCN-Al complex, the interaction becomes markedly unidirectional. Charge transfer from the nanocarrier to TMZ remains weak (*E*^2^ < 0.37 kcal mol^−1^). In contrast, several TMZ → gCN-Al transitions exhibit significantly higher stabilization energies, notably LP(2)N60 → π*(C22–N29) and LP(1)C65 → π*(C1–N10) with *E*^2^ values of 1.49 and 1.27 kcal mol^−1^, respectively. Furthermore, donor–acceptor interactions from TMZ nitrogen lone pairs to the Al atom (TMZ → Al) are observed, albeit with low stabilization (*E*^2^ ≈ 0.03–0.04 kcal mol^−1^). This indicates the role of Al as an electron-accepting site, strengthening the donor–acceptor character of the interaction and enhancing adsorption stability. Conversely, the TMZ@gCN-Ga complex exhibits minimal charge transfer in all directions, with the highest *E*^2^ value being only 0.14 kcal mol^−1^. The weak orbital interactions suggest that the stability of this complex is not primarily governed by significant donor–acceptor CT. Instead, its strong adsorption, as indicated by the adsorption energy, is likely stabilized by other non-covalent forces, such as van der Waals interactions and electrostatic complementarity, as identified in NCI analyses.

### 3.6 TD-DFT simulated UV-visible absorption spectra

**Fig. 7:**
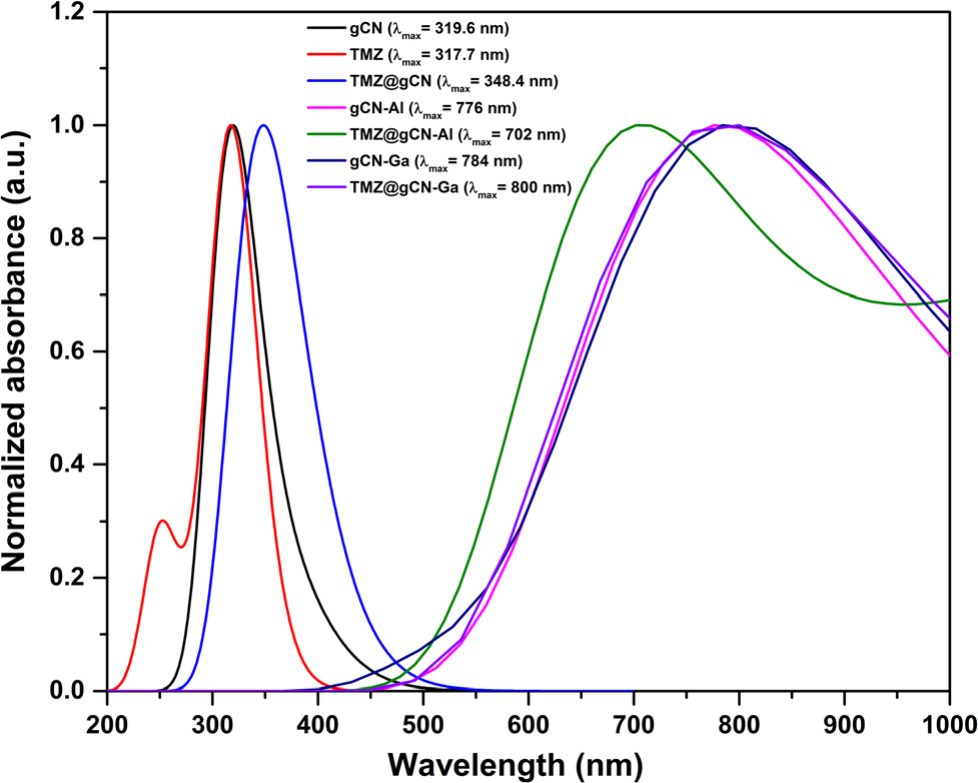
TD-DFT simulated UV-vis absorption spectra of TMZ, gCN, TMZ@gCN, gCN-Al, TMZ@gCN-Al, gCN-Ga, and TMZ@gCN-Ga.

Fig. 7 shows the normalized UV-vis spectra of TMZ, gCN, its doped variants (gCN-Al and gCN-Ga) and their complexes. gCN had a maximum absorption wavelength (*λ*_max_) of 319.6 nm. Following that, the TMZ@gCN complex exhibits a maximum absorption wavelength (*λ*_max_) of 348.4 nm, which is red-shifted (shifted to a longer wavelength) compared to the 317.7 nm (*λ*_max_) of the TMZ and the optical band gap (*ε*_gap_) energy is approximately equivalent to the energy of photons absorbed at its maximum absorption wavelength (*λ*_max_) of 348.4 nm. This bathochromic shift is consistent with the reduced HOMO–LUMO gap (*ε*_gap_) in TMZ@gCN. A smaller energy gap between electronic states results in the absorption of lower-energy photons, which correspond to longer wavelengths. Elemental doping with Al and Ga markedly alters the electronic properties, generating maximum absorption spectra at 776 nm and 784 nm respectively which corroborated to the reduced HOMO–LUMO gap (*ε*_gap_). Similar to TMZ@gCN, red shifting is also observed in TMZ@gCN-Ga (*λ*_max_ = 800 nm) compared to the bare gCN-Ga (*λ*_max_ = 784 nm). However, TMZ@gCN-Al exhibits a maximum absorption wavelength (*λ*_max_) of 702 nm, a blue-shifted absorption spectrum from its bare counterpart, gCN-Al (*λ*_max_ = 776 nm).

### 3.7 Calculated infrared spectral features

**Fig. 8:**
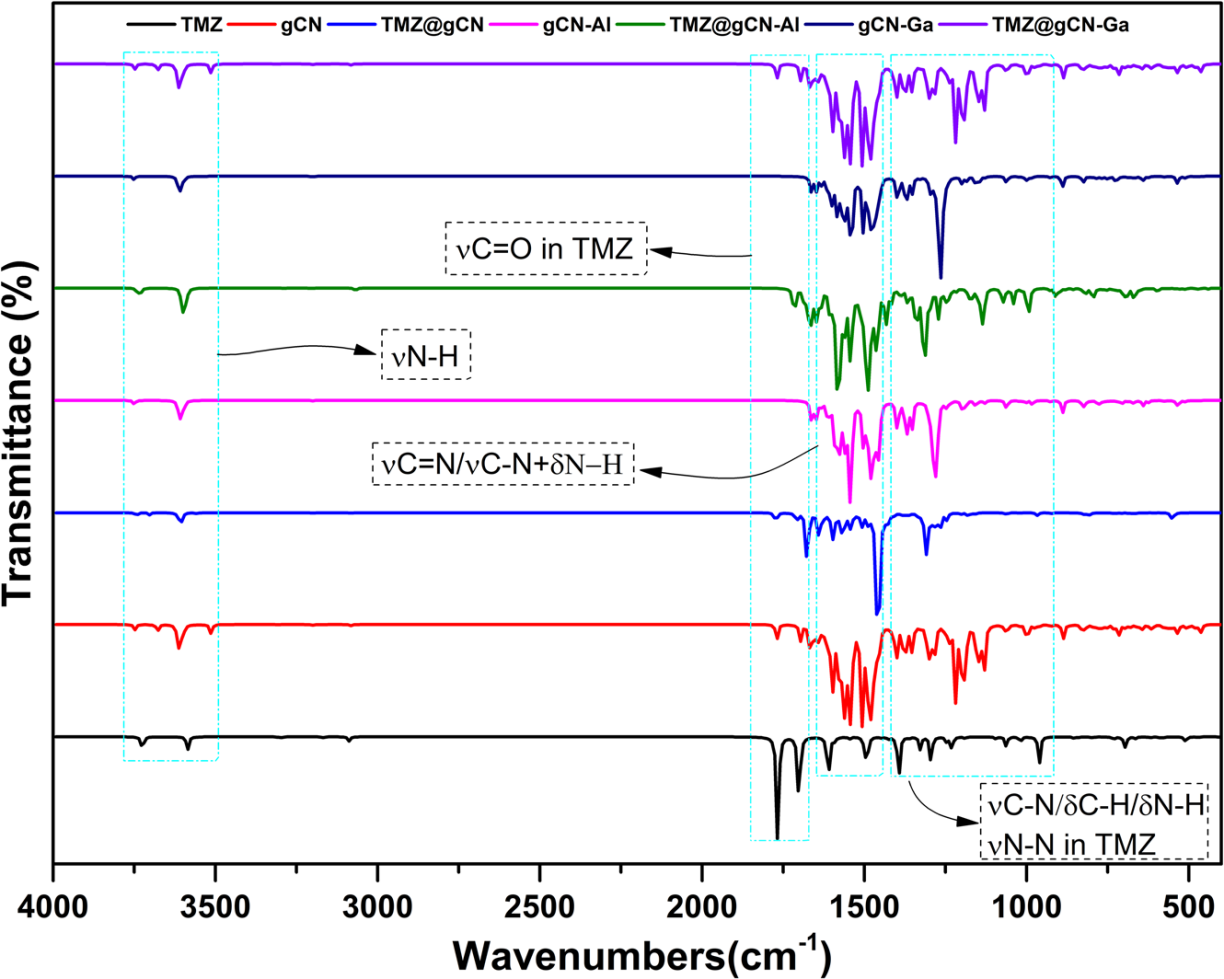
DFT-calculated FTIR spectra of TMZ, gCN, Al/Ga-modified gCN and their complexes showing characteristic vibrational bands.

IR calculation revealed no imaginary frequency for the complexes confirming their true minimum state of the stable optimized geometry. IR spectra (Fig. 8) revealed the formation of the TMZ@gCN complex as evidenced by significant alterations in peak intensities and the appearance of new vibrational modes, indicating drug–nanocarrier interaction. Notably, Al/Ga doping further modified the IR spectra. Observed peak shifts indicate a change in bond strength and electron density within the nanocarrier due to the dopant atoms. Concurrent alterations in peak intensity and peak shifting upon TMZ adsorption to the carrier surface suggest enhanced intermolecular forces, such as stronger hydrogen bonding or charge-transfer interactions.^71,72^ These spectral changes are consistent with noncovalent complex formation between TMZ and the nanocarriers.

### 3.8 Noncovalent interaction (NCI) mapping

The nature and spatial distribution of the intermolecular forces responsible for stabilizing the TMZ with the nanocarriers were elucidated through Non-Covalent Interaction (NCI) analysis. This approach, grounded in the topology of the electron density and its reduced gradient (RDG), provides a visual and quantitative map of the weak forces that are central to physisorption processes.^73^ The resulting plots, which graph the RDG against sign(*λ*_2_)*ρ* reveal the characteristic signatures of different interaction types: hydrogen bonds appear as spikes in the negative sign(*λ*_2_)*ρ* region, van der Waals forces cluster near zero, and steric repulsion is indicated by features at positive values.

**Fig. 9:**
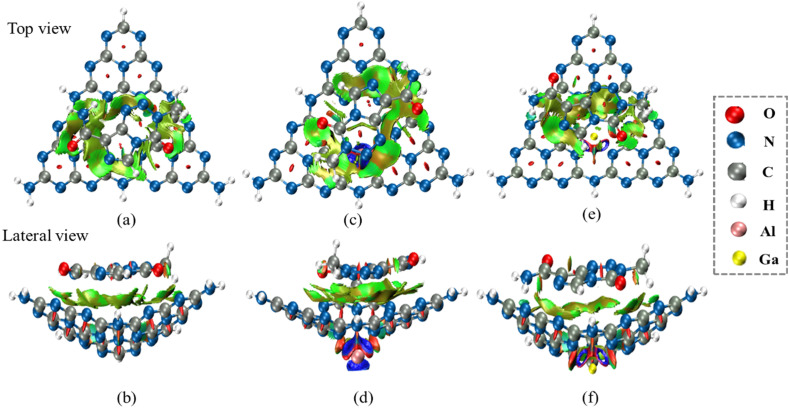
3D iso-surfaces of (a and b) TMZ@gCN, (c and d) TMZ@gCN-Al, and (e and f) TMZ@gCN-Ga.

The NCI iso-surfaces (Fig. 9) for all TMZ@nanocarrier complexes consistently show extended green discs located between the drug and carrier interfaces. These features signify the presence of favorable, weak-to-medium strength non-covalent interactions, primarily van der Waals forces and weak hydrogen bonds, which primarily stabilize the TMZ@nanocarrier complexes. A comparative examination, however, reveals a distinct evolution in the interaction profile upon doping.

**Fig. 10:**
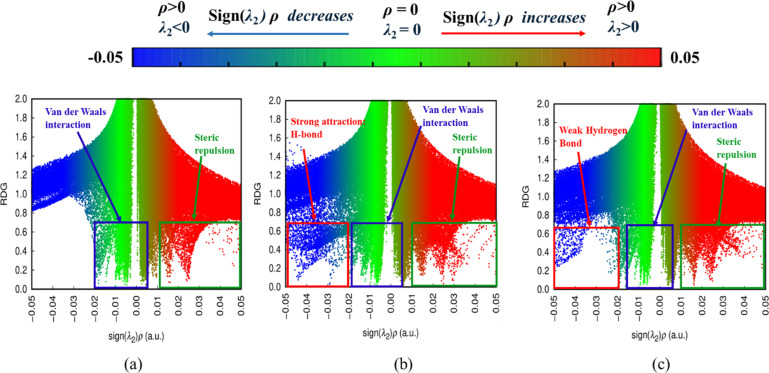
2D RDG plots of (a) TMZ@gCN, (b) TMZ@gCN-Al, and (c) TMZ@gCN-Ga. The color bar represents sign(*λ*)*ρ*(*r*) in atomic units.

The 2D scatter plot (Fig. 10(b and c)) of TMZ@gCN-Al and TMZ@gCN-Ga complexes shows a pronounced density of green points in the region where sign(*λ*_2_)*ρ* is close to zero. This indicates that van der Waals interactions contribute to the stabilizing force in these systems. Again, the RDG scatter plot exhibits a higher density of blue points extending further into the negative sign(*λ*_2_)*ρ* region (approximately −0.05 to −0.02 a.u.). This pattern is indicative of stronger, more directional hydrogen bonds, such as O⋯H and N⋯H. However, the absence of pronounced blue spikes for TMZ@gCN (Fig. 10(a)) suggests that strong directional hydrogen bonding is limited in the pristine complex, although weak N⋯H contacts are still indicated by the optimized geometry and QTAIM bond paths. The non-covalent nature of these specific interactions facilitates reversible binding. These reversible forces are susceptible to disruption by the competitive solvation or changes in the local environment at the target site, thereby facilitating the controlled release of the TMZ payload.

Overall, NCI mapping suggests that TMZ@gCN is mainly stabilized by van der Waals contacts and weak N⋯H interactions, whereas the Al- and Ga-doped complexes show stronger contributions from directional hydrogen-bond-like contacts together with van der Waals interactions.

### 3.9 Topological analysis of electronic interactions (QTAIM)

**Fig. 11:**
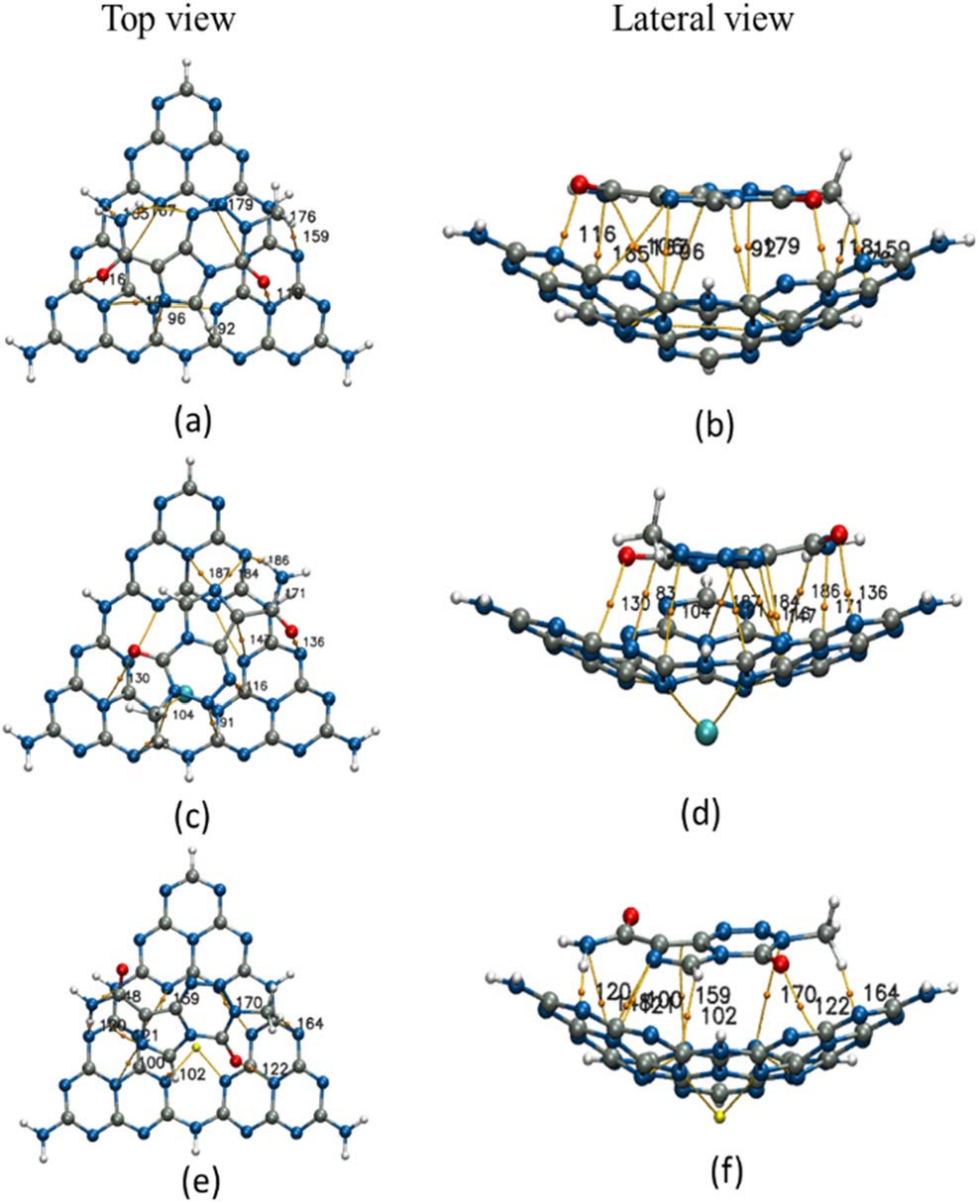
Key BCPs of TMZ@gCN (a and b), TMZ@gCN-Al (c and d) & TMZ@gCN-Ga (e and f) complexes, along with the bond-paths (orange lines) and bond critical points: orange circles, representing the possible bonding interactions. The bond paths were calculated from Bader's QTAIM analysis.

The nature and strength of the intermolecular interactions in the TMZ@nanocarrier complexes were calculated and visualized using the Quantum Theory of Atoms in Molecules (QTAIM) at each bond critical point (Table 4 and Fig. 11). The topological parameters presented in Table 4 provide definitive criteria to classify bonding character.^52^

**Table 4:** The electron density (*ρ*(*r*)), Laplacian of electron density (∇^2^*ρ*(*r*)), electron kinetic energy density *G*(*r*), electron potential energy density *V*(*r*), electron Hamiltonian energy density *H*(*r*), and bond ellipticity (*ε*) values at the bond critical points (BCPs) of the TMZ/nanocarrier interface

Complex	BCP	Interaction	*ρ*(*r*)	∇^2^*ρ*(*r*)	*V*(*r*)	*G*(*r*)	*G*(*r*)/|*V*(*r*)|	*H*(*r*)
TMZ@gCN	92	67(H)⋯33(C)	0.0046	0.0178	−0.0022	0.0033	1.5000	0.0011
	96	22(C)⋯57(N)	0.0057	0.0191	−0.0033	0.0040	1.2121	0.0008
	106	57(N)⋯25(N)	0.0045	0.0154	−0.0029	0.0034	1.1724	0.0005
	116	54(O)⋯19(C)	0.0098	0.0370	−0.0068	0.0080	1.1765	0.0001
	118	53(O)⋯40(N)	0.0010	0.0332	−0.0072	0.0078	1.0833	0.0005
	159	39(N)⋯69(H)	0.0083	0.0257	−0.0048	0.0056	1.1667	0.0008
	165	60(N)⋯16(N)	0.0057	0.0156	−0.0036	0.0037	1.0278	0.0002
	167	60(N)⋯13(N)	0.0042	0.0134	−0.0025	0.0030	1.2000	0.0004
	171	58(N)⋯13(N)	0.0065	0.0203	−0.0041	0.0046	1.1220	0.0005
	176	69(H)⋯15(N)	0.0054	0.0193	−0.0032	0.0040	1.2500	0.0008
TMZ@gCN-Al	83	71(H)⋯43(N)	0.0097	0.0320	−0.0056	0.0068	1.2143	0.0012
	91	22(C)⋯60(N)	0.0123	0.0371	−0.0069	0.0081	1.1739	0.0012
	104	33(C)⋯57(N)	0.0063	0.0211	−0.0038	0.0045	1.1842	0.0008
	116	59(N)⋯21(C)	0.0096	0.0313	−0.0057	0.0068	1.1930	0.0011
	130	40(N)⋯54(O)	0.0078	0.0253	−0.0055	0.0060	1.0909	0.0004
	136	55(O)⋯23(N)	0.0077	0.0235	−0.0051	0.0055	1.0784	0.0004
	147	24(N)⋯63(C)	0.0067	0.0177	−0.0035	0.0040	1.1429	0.0005
	171	61(N)⋯16(N)	0.0077	0.0205	−0.0048	0.0050	1.0417	0.0002
	184	58(N)⋯11(N)	0.0069	0.0206	−0.0042	0.0047	1.1190	0.0005
	186	72(H)⋯11(N)	0.0094	0.0313	−0.0058	0.0068	1.1724	0.0010
	187	58(N)⋯10(N)	0.0077	0.0225	−0.0048	0.0052	1.0833	0.0004
TMZ@gCN-Ga	100	40(N)⋯58(N)	0.0058	0.0189	−0.0038	0.0043	1.1316	0.0005
	102	68(H)⋯41(N)	0.0062	0.0206	−0.0032	0.0042	1.3125	0.0001
	120	39(N)⋯72(H)	0.0188	0.0521	−0.0126	0.0128	1.0159	0.0002
	121	58(N)⋯35(C)	0.0079	0.0256	−0.0047	0.0056	1.1915	0.0008
	122	25(N)⋯54(O)	0.0093	0.0343	−0.0067	0.0076	1.1343	0.0009
	148	61(N)⋯15(N)	0.0061	0.0171	−0.0038	0.0040	1.0526	0.0002
	159	62(C)⋯14(N)	0.0093	0.0283	−0.0050	0.0061	1.2200	0.0010
	164	23(N)⋯69(H)	0.0119	0.0347	−0.0073	0.0080	1.0959	0.0007
	170	57(N)⋯13(N)	0.0076	0.0230	−0.0046	0.0052	1.1304	0.0006

The QTAIM parameters are consistent with predominantly noncovalent interactions. This is evidenced by the low values of electron density (*ρ*(*r*) < 0.02 a.u.) and the positive Laplacian (∇^2^*ρ*(*r*) > 0) at all identified BCPs, which are hallmark features of interactions where electron density is depleted in the internuclear region. The ratio of the kinetic energy density to the absolute value of the potential energy density, *G*(*r*)/|*V*(*r*)|, further substantiates this finding. For all critical interactions, this ratio is greater than 1, which is characteristic of closed-shell (non-covalent) interactions such as hydrogen bonding and van der Waals contacts, as opposed to shared (covalent) interactions where the ratio is typically less than 0.5.^74^

The strength of these non-covalent interactions varies significantly across the complexes, correlating with the adsorption energy trend. The QTAIM analysis reveals a stronger non-covalent interaction in the TMZ@gCN-Ga complex compared to the TMZ@gCN-Al system, as quantified by the electron density at key bond critical points (BCPs). In the Ga-doped complex, the N–H interactions between TMZ and gCN-Ga are characterized by substantial electron densities, with *ρ*(*r*) = 0.0188 a.u. for the 39(N)⋯72(H) bond (BCP 120) and *ρ*(*r*) = 0.0119 a.u. for the 23(N)⋯69(H) bond (BCP 164). This contrasts with the TMZ@gCN-Al complex, where only one BCP, corresponding to the 22(C)⋯60(N) interaction (BCP 91), exhibits a comparable electron density in the hundredths range (*ρ*(*r*) = 0.0123 a.u.). The significantly higher *ρ*(*r*) value for the primary N–H interaction in the TMZ@gCN-Ga complex (0.0188 a.u.) reflects a stronger hydrogen bond. This is further corroborated by a more negative potential energy density, *V*(*r*) = −0.0126 a.u., which indicates greater stabilization energy at this interface. The combination of these topological parameters provides a quantitative basis for the increased adsorption energy and the closest distance observed for the H72⋯N39 atom pair in TMZ@gCN-Ga.

In summary, QTAIM analysis provides topological support for the predominantly noncovalent nature of TMZ adsorption. The data quantitatively rationalize the enhanced adsorption in the doped systems, TMZ@gCN-Al and TMZ@gCN-Ga, by revealing stronger and more concentrated electron density distributions at the critical points of interaction, consistent with the calculated adsorption energies.

### 3.10 Estimated recovery time

The recovery time (*τ*), a parameter describing the characteristic desorption period of a drug from its carrier surface, is a critical determinant of release kinetics in nanoscale drug delivery systems. For the complexes, the measured *τ* values span several orders of magnitude, revealing a temperature dependent trend (Table 5). As temperature increases from 298.15 K to 315.5 K, *τ* decreases significantly for all three complexes, a trend consistent with an Arrhenius-type, thermally activated release process. This direct relationship aligns with theoretical studies where elevated temperatures provide the kinetic energy needed to overcome the activation barrier for drug desorption.^53^

**Table 5:** Recovery time (*τ*, in s) for the complexes

Complexes	*T* (298.15 K)	*T* (310.15 K)	*T* (315.5 K)
TMZ@gCN	7.42 × 10^−4^	1.97 × 10^−4^	1.17 × 10^−4^
TMZ@gCN-Al	2.46 × 10^−2^	5.71 × 10^−3^	3.21 × 10^−3^
TMZ@gCN-Ga	8.99 × 10^2^	1.39 × 10^2^	6.66 × 10^1^

The nanocarrier composition profoundly influences the drug's retention, with the undoped TMZ@gCN complex exhibiting the shortest *τ* (on the order of 10^−4^ s), indicating a short retention suitable for rapid release. Doping with Al extends *τ* to the 10^−3^ to 10^−2^ s range, suggesting a moderate retention period, while Ga doping results in the longest *τ* (10^1^ to 10^2^ s), implying a higher retention and potential for prolonged, sustained release. This trend shows a positive correlation between adsorption energy and recovery time, where stronger binding leads to longer desorption periods.

From a therapeutic perspective, this spectrum of *τ* values enables the design of tailored release profiles. The fast-release profile of TMZ@gCN could be advantageous for acute treatments requiring immediate drug availability. In contrast, the extended *τ* of TMZ@gCN-Ga makes it a candidate for long-circulating or depot systems, potentially reducing dosing frequency and minimizing off-target effects. The pronounced temperature sensitivity of all complexes is particularly promising for developing stimuli-responsive systems. Localized hyperthermia, a clinically established method for tumor treatment, could be used to trigger rapid, on-demand TMZ release at the target site, a strategy actively explored in smart thermosensitive nanocarrier design.^75^

### 3.11 Dipole moment and interfacial polarization

The dipole moments revealed distinct electronic polarization upon doping and drug adsorption (Table 6). Pristine gCN (6.78 D) shows increased polarization upon TMZ adsorption (7.51 D), indicating interfacial charge redistribution. Al-doping further amplifies this effect: the TMZ@gCN-Al complex exhibits a markedly elevated dipole moment (11.61 D), indicating strong donor–acceptor interaction and significant electron density asymmetry at the interface. This contrasts with the more modest increase in the Ga-doped analogue (8.66 D).

**Table 6:** Dipole moment of all the studied systems

System	Dipole moment (D)
TMZ	5.07
gCN	6.78
TMZ@gCN	7.51
gCN-Al	5.20
TMZ@gCN-Al	11.61
gCN-Ga	3.70
TMZ@gCN-Ga	8.66

The pronounced dipole moment in TMZ@gCN-Al suggests a stronger, more polar drug–carrier interaction. This enhanced polarity can facilitate environment-responsive release;^76^ the electrostatic interface may be more susceptible to dissociation triggered by local pH changes or ionic gradients in biological microenvironments. Furthermore, increased molecular polarity correlates with improved aqueous solubility, a key determinant of the dissolution rate and bioavailability.^77^

### 3.12 Thermodynamic profile

The computed thermodynamic parameters (Table 7) for the TMZ@nanocarrier complexes reveal a consistent pattern of spontaneous, exothermic binding, with significant variations driven by carrier doping. The Gibbs free energy of binding (Δ*G*) is markedly more negative for TMZ@gCN-Ga (−56.19 kcal mol^−1^) and TMZ@gCN-Al (−23.32 kcal mol^−1^) than for the undoped variant, TMZ@gCN (−20.17 kcal mol^−1^), indicating stronger complex stability for the doped systems, particularly TMZ@gCN-Ga. This increase in thermodynamic favorability is enthalpy-driven, as reflected in the substantially larger negative Δ*H* value for TMZ@gCN-Ga (−111.18 kcal mol^−1^) and TMZ@gCN-Al (−84.79 kcal mol^−1^), which points to the formation of stronger noncovalent stabilization, arising from a combination of electrostatic polarization, hydrogen-bond-like contacts, and dispersion interactions. The small, negative entropy change (Δ*S*) associated with complex formation is consistent with a modest loss of conformational freedom upon drug adsorption, a typical feature of surface-confined binding.

**Table 7:** Computed thermodynamic parameters (kcal mol^−1^) for TMZ@nanocarrier complexes

Complexes	Δ*G*	Δ*H*	Δ*S*
TMZ@gCN	−20.17	−75.56	−0.19
TMZ@gCN-Al	−23.32	−84.79	−0.21
TMZ@gCN-Ga	−56.19	−111.18	−0.18

The increased Δ*G* and Δ*H* values upon Ga and Al-doping underscore the important role of metal doping in tailoring the electronic and surface characteristics of graphitic carbon nitride for optimal drug–carrier interaction. The more negative thermodynamic parameters of the doped complexes suggest stronger TMZ retention within the present model. Whether this translates into higher loading or retention in real nanocarrier systems requires experimental validation and simulations using larger, solvated carrier models.

## 4 Conclusion

This DFT study provides molecular-level insight into the adsorption of temozolomide on pristine and Al/Ga-doped graphitic carbon nitride fragments. The calculated adsorption energies indicate that metal doping strengthens TMZ binding, following the order gCN-Ga > gCN-Al > pristine gCN. NCI and QTAIM analyses suggest that the complexes are stabilized predominantly by noncovalent interactions, including van der Waals contacts and weak-to-moderate hydrogen-bonding interactions. Electronic-structure analyses show that Al/Ga doping narrows the molecular HOMO–LUMO gap and increases interfacial polarization, although the role of charge transfer differs between Al- and Ga-doped systems. Recovery-time estimates suggest that TMZ desorption is thermally accessible, with Ga doping producing the longest predicted residence time. Overall, the results indicate that dopant engineering can tune TMZ–gCN interactions and provide a useful computational basis for future experimental and higher-level theoretical studies on gCN based drug delivery platforms. However, biological delivery performance, BBB transport, toxicity, and stimulus-responsive release require direct experimental validation beyond the present finite-cluster DFT model.

## Author contributions

Maisha Yousuf: investigation, data curation, visualization, formal analysis, software, writing – original draft, writing – review & editing. Mohammed Sakib Musa: conceptualization, project administration, software, methodology, investigation, formal analysis, data curation, validation, writing – original draft, writing – review & editing. Arafat Mahamud Bhuiyan: resources, software, writing – review & editing. Monir Uzzaman: supervision, software, writing – review & editing. Md. Moazzam Hossain: supervision, writing – review & editing. Kamol Dey: supervision, writing – review & editing.

## Conflicts of interest

The authors declare no competing interests.

## Supplementary Material

NA-OLF-D6NA00001K-s001

## Data Availability

Data are provided within the manuscript or supplementary information (SI). Supplementary information is available. See DOI: https://doi.org/10.1039/d6na00001k.
