# Supplementary material for: Al- and Ga-doped graphitic carbon nitride as a temozolomide nanocarrier platform: a DFT study of adsorption and interfacial interactions
Source: Nanoscale Adv. 2026 Jun 5;8(14):4109–23. doi: 10.1039/d6na00001k (PMC13277665; doi:10.1039/d6na00001k)
Supplement: NA-008-D6NA00001K-s001 [file NA-008-D6NA00001K-s001.pdf]

Supplementary Information (SI) for Nanoscale Advances.
This journal is © The Royal Society of Chemistry 2026

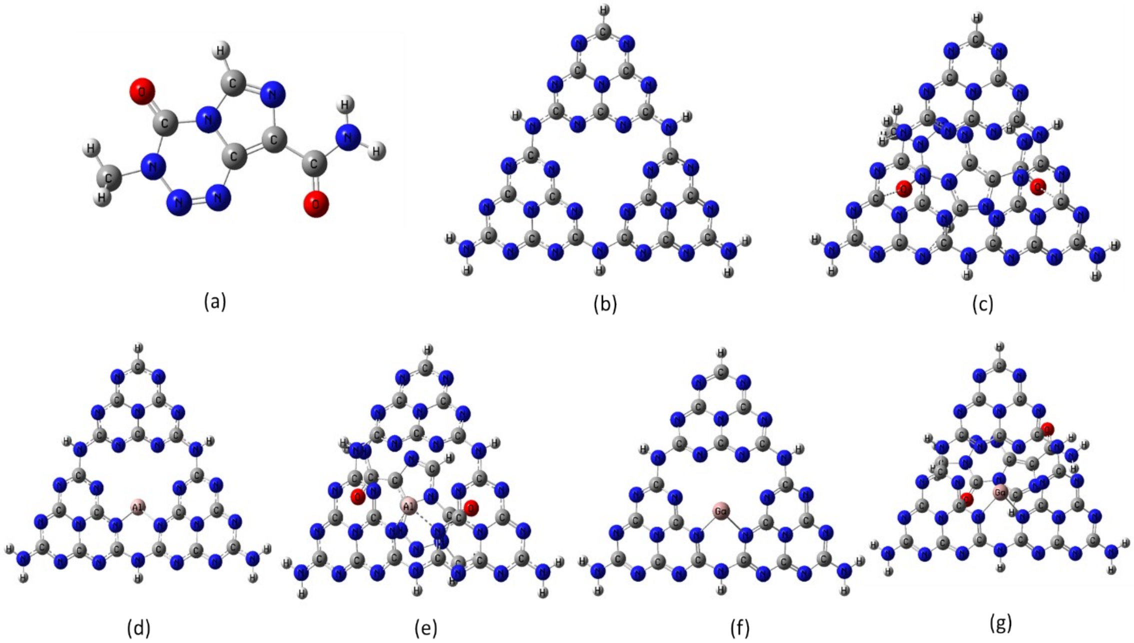


**Figure S1:** Top view of the optimized structures of a) TMZ, b) gCN, c) TMZ@gCN, d) gCN-Al, e) TMZ@gCN-Al, f) gCN-Ga, and g) TMZ@gCN-Ga

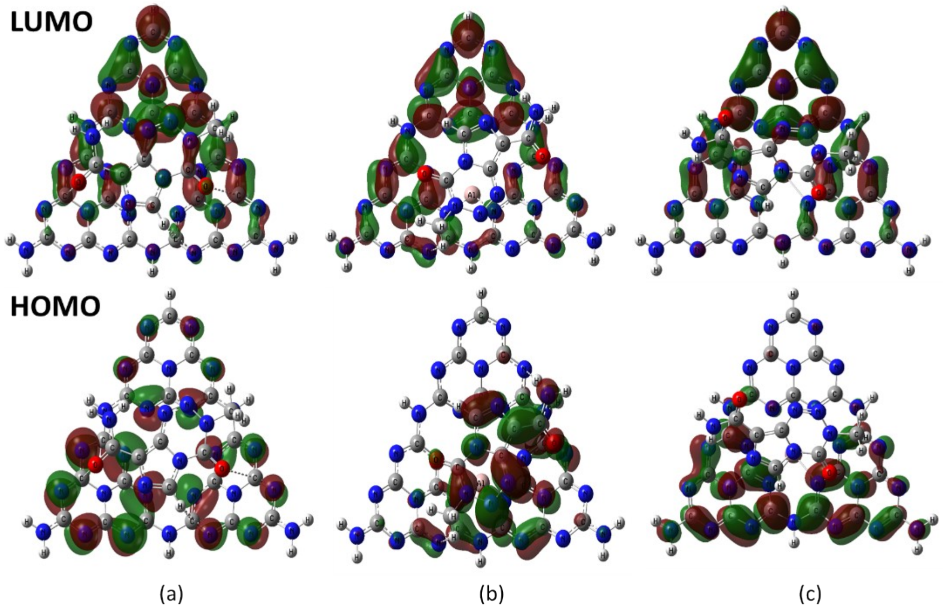


**Figure S2:** Top view of HOMO-LUMO orbitals of (a) TMZ@gCN, (b) TMZ@gCN-Al, and (c) TMZ@gCN-Ga
